# Archimedean Spiral Pairs with no Electrical Connections as a Passive Wireless Implantable Sensor

**Published:** 2014-08-18

**Authors:** John F Drazan, Aleksandra Gunko, Matthew Dion, Omar Abdoun, Nathaniel C Cady, Kenneth A Connor, Eric H Ledet

**Affiliations:** 1 Rensselaer Polytechnic Institute, Troy, NY; 2 State University of New York College of Nanoscale Science and Engineering, Albany, NY

**Keywords:** Sensor, Passive Resonator, Wireless, Implantable

## Abstract

We have developed, modeled, fabricated, and tested a passive wireless sensor system that exhibits a linear frequency-displacement relationship. The displacement sensor is comprised of two anti-aligned Archimedean coils separated by an insulating dielectric layer. There are no electrical connections between the two coils and there are no onboard electronics. The two coils are inductively and capacitively coupled due to their close proximity. The sensor system is interrogated wirelessly by monitoring the return loss parameter from a vector network analyzer. The resonant frequency of the sensor is dependent on the displacement between the two coils. Due to changes in the inductive and capacitive coupling between the coils at different distances, the resonant frequency is modulated by coil separation. In a specified range, the frequency shift can be linearized with respect to coil separation. Batch fabrication techniques were used to fabricate copper coils for experimental testing with air as the dielectric. Through testing, we validated the performance of sensors as predicted within acceptable errors. Because of its simplicity, this displacement sensor has potential applications for in vivo sensing.

## Introduction

Recent trends in healthcare have placed an emphasis on cost savings. For patients undergoing surgery, strategies which facilitate shorter recovery times, result in fewer complications, and require fewer revisions can affect substantial cost reductions. Intra-operative and post-operative decisions guided by patient-specific data can lead to optimized personalized treatment regimens. Objective real-time quantitative patient-specific data after surgery can provide critical information on the progression of recovery or healing of a patient. These data can be used to customize care and influence the rehabilitation regimen on a patient-specific basis.

In orthopaedic surgery and neurosurgery, implantable sensors are a potential enabling technology for obtaining patient-specific data. Sensors can be incorporated into the implants which are often used in these procedures [1]. Post-operatively, the sensors can provide objective quantitative data informing the healthcare team about the progression of healing (or lack thereof). This can guide post-operative care and rehabilitation therapy which can improve outcomes and reduce recovery time. There are many applications in orthopaedic surgery where implantable sensing technology has the potential to enable these cost savings including joint replacements [2], fracture fixation [3], and spine surgery [4]. For example, early detection of a fracture non-union can be achieved by measuring load sharing between the implant and bone following surgery [1].

Sensor-enabled orthopaedic implants were established decades ago. Despite this long history, they have yet to be applied in the clinical setting. There continue to be significant impediments to the progress of sensor-enabled orthopaedic implants. The major challenges continue to be (a) compatibility with existing implants, (b) cost, and (c) longevity within the human body [1,5].

### Compatibility with Existing Implants

Implant manufacturers expend extensive time and capital to develop and test implants for clinical use. Implantable sensing systems which require extensive post-production alteration to implants introduce additional manufacturing costs and regulatory hurdles for the sensor-instrumented implants.

### Cost

In 2010, internal fixation of fractures, lumbar fusions, total knee replacements, and total hip replacements were performed 1.8 million times in the United States alone [6]. The ubiquity of orthopaedic procedures makes any significant per unit increase in cost a substantial burden on the healthcare system. Previous sensor systems have required expensive signal conditioning electronics and telemetry or complex MEMS systems, all of which add substantial costs.

### Longevity within the Human Body

One of the most significant challenges in long term sensing is protection of the electronics from the environment in the body. Long term implants such as pacemakers are packaged in hermetically sealed metal cans. Because of the bulk of this packaging, alternative strategies such as conformal coating are attractive for orthopaedic smart implants, but not as robust. Protection from the in vivo environment remains a significant challenge, particularly for sensing systems which require signal conditioning electronics.

To surmount these challenges, we have taken an alternative approach to in vivo sensing. We have developed a simple, passive, wireless implantable sensing system which is small enough to obviate the need for modification of implants, inexpensive enough to introduce negligible increase in cost, and simple enough that it is robust in the in vivo environment. The sensor consists only of two anti-aligned Archimedean spirals with a dielectric between [7]. There are no other components to the sensor. Radiofrequency communication via an external antenna facilitates wireless monitoring. As described below, the sensors function as simple passive resonators. They transduce a change in distance between the two Archimedean spirals into a measureable change in resonant frequency. Using this simple relationship, they can be used to transduce various physical changes in the sensor's environment such as force, pressure, or temperature. The purpose of this work was to develop the operating principles of this new class of sensor, describe its linear frequency-displacement relationship over specific ranges, and validate it as a displacement transducer experimentally.

## Materials and Methods

### Sensor Description

**Figure 1:**
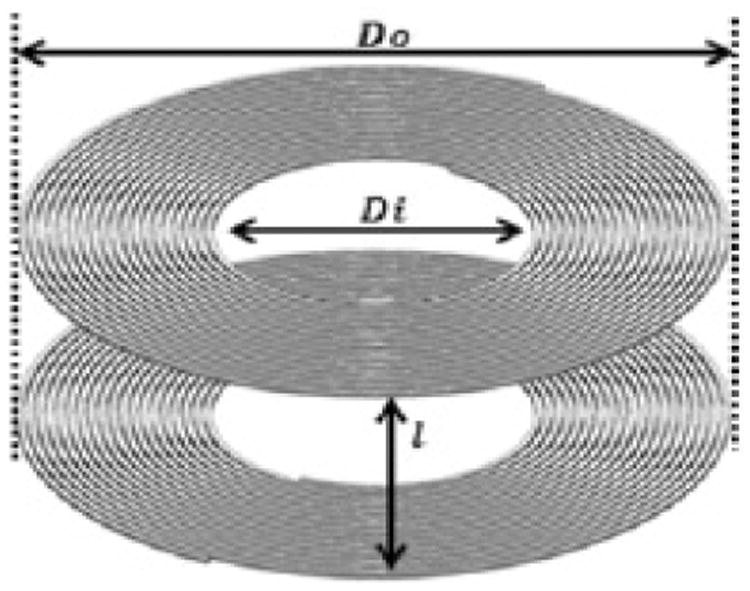
A sensor is comprised of paired coils with a dielectric between. The outer diameter is D_o, the inner diameter D_i, and the spacing between coils is l.

**Figure 2:**
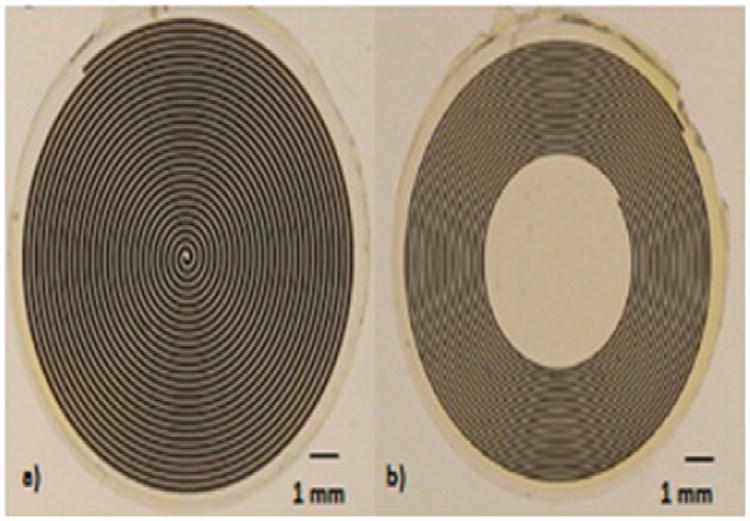
Coils were fabricated using microfabrication techniques in various configurations and sizes including disk coils (a) and halo coils (b) depending on the application.

**Figure 3:**
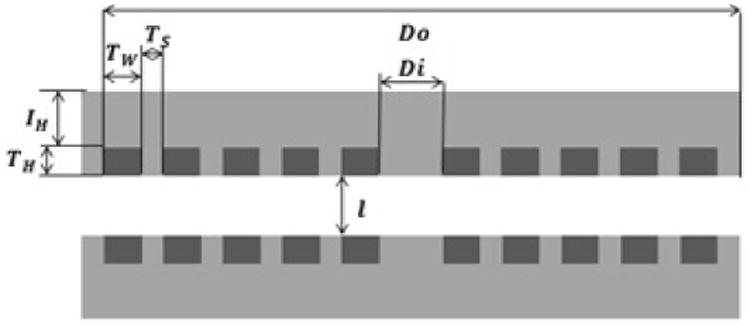
Sensor geometry is defined by its cross section. Geometric properties of the conductive trace are defined by the trace spacing (T_S), width (T_w) and height (T_H). The overall sensor geometry is determined by the inner diameter (D_i), outer diameter, (D_o) and coil displacement (l). Each coil is embedded in a layer of KMPR photoresist that is defined by a set insulator height (I_H).

The sensor system consists of two anti-aligned Archimedean coils as shown in Figure 1. Here we describe a “halo” or ring coil, although the coils can take on any shape or size including a disk (Figure 2). Between the two coils is a dielectric. The overall geometry of the round coils is defined by the inner diameter (Di), the outer diameter (Do), number of turns (N) and the coil separation (l). Each coil is also defined by its cross sectional geometry as illustrated in Figure 3. The trace height, width and spacing are defined as T_H, T_W, and T_S respectively. Each coil is embedded in an insulating material with a top layer defined by the insulator height variable I_H. Representative geometries properties for a large halo coil are shown in Table 1.

The simple displacement sensor is formed by anti-aligning two coils and separating them with a dielectric. The spacing between the coils or thickness of the dielectric (l) can be varied. A change in spacing modulates the electrical characteristics of the sensor as described below. For the dimensions shown in Table 1, the upper limit for spacing between the coils is approximately 1 cm.

### Model of sensor Behavior

The flat, parallel, paired Archimedean coils act as both inductor and capacitor making this simple configuration an inductor-capacitor (LC) resonator [8–10]. When exposed to an external radiofrequency (RF) field, the sensor resonates. The resonant frequency can be monitored wirelessly using the return loss parameter as measured by a network analyzer. This serves as the means of monitoring the sensor [8,11, 12].

The resonant frequency is dependent on several parameters related to the sensor's geometry and material properties. We have developed an analytical relationship between the geometry of the coils and the sensor resonant frequency using a lumped constant LC model [8, 13]. We have assumed that there is inductive and capacitance coupling between the coils and also that each spiral has distributed capacitance and inductance. These discrete contributions are lumped in the general relationship to model the resonant frequency of a series LC circuit:

(1) $$f_{0}=1/(2\pi \surd LC)$$

In this relationship, f_0 is the resonant frequency, L is the total inductance, and C is the total capacitance.

This sensor operates on the assumption that changes in the resonant frequency are caused solely by changes in the spacing between the coils. This relationship is developed in detail below and provides a means by which coil spacing can be determined from a simple frequency measurement. In this manner the sensor functions as a wireless displacement sensor through the monitoring of the changes in resonant frequency. To determine coil spacing from frequency, the total inductance and total capacitance in Equation 1 are derived analytically below based on the geometry and dielectric properties of the components with the exception of the parasitic capacitance which is measured through a simple calibration procedure.

### Total Inductance

The lumped inductance is the sum of a single coil's inductance and the mutual inductance between the two coils. The inductance of a single coil remains constant regardless of coils spacing. Thus changes in mutual inductance must be responsible for changes in total inductance. We have utilized a model based on the current sheet approximation with empirical corrections to model the inductance of a single circular coil [14].

(2) $$L_{\text{Coil}}=(\mu_{0}N^{2}D_{\text{avg}}c_{1})/2(\ln(c_{2}/\rho )+c_{3}\rho +c_{4}\rho^{2})$$

Where ρ is the fll factor= (D_o_-D_i_)/(D_o_+D_i_), D_avg_ is the average diameter of the coil= (D_o_+D_i_)/2 and c1 through c4 are constants related to the circular geometry of the coil. For a circular planar spiral as shown in Figure 1, the constants are 1.0, 2.46, 0.0 and 0.02 respectively. This relationship accounts for only a single isolated coil so it is necessary to take into account the effects of the opposing coil on the inductance of the entire system.

Mutual inductance represents the interaction between the magnetic fields generated by the current in each of the coils. Commonly, the mutual inductance between two aligned coaxial coils is calculated by treating each turn in a coil as a conductive ring [15–18]. The total mutual inductance of the system can be found by calculating the partial inductance of every turn in one coil with respect to every turn in the opposite coil using Equations 3-5. These contributions are summed using Equation 6 [16].

(3) $$M\_\text{ij}=(2\mu_{r}\mu_{0})/\alpha \surd (r\_i\cdot r\_j)[(1-\alpha^{2}/2)K(m)-E(m)]$$

(4) $$m={\left(\sin\alpha \right)}^{2}$$

(5) $$\alpha =2\surd ((r_{i}\cdot r_{j})/({\left(r_{i}+r_{j}\right)}^{2}+l^{2}))$$

(6) $$M=\sum_{\left(i=1\right)}^{\left(N^{1}\right)}\sum_{\left(i=1\right)}^{\left(N^{2}\right)}M_{ij}(r_{i}.r_{j},l)$$

In these equations, N_1 and N_2 are the number of turns in each coil. In the case of matched paired coils, they are equal. l is the separation between the coils. μ_r is the relative permeability of the dielectric media between the coils and μ_0 is the permeability of free space. K(m) and E(m) are complete elliptic integrals of the 1st and 2nd kind respectively while m and α are the elliptic parameter and modular angle, respectively. These are based on the geometry of the coils and are inputs to the elliptic integrals. To model the mutual inductance between the spirals, each turn is treated as a conductive ring [15]. That is, a 19 turn coil is modeled as 19 concentric rings whose radii are defined by the distance ri and rj, where ri is the radius of the top ring and rj is the radius of the bottom ring.

The total mutual inductance M between two coils from Equation 6 can be normalized to the inductance of a single coil Lcoil to define the coupling constant k.

(7) $$k=M/L_{\text{Coil}}$$

In our novel sensors, there are no electrical connections between the two coils. This distinguishes our sensors relative to other systems which incorporate one or more viasconnecting the coils [8,11]. At large coil separations, the resonant behavior of our sensors shifts to that of a single isolated coil because there are no electrical connections.

Thus, at large separations the total sensor inductance is dominated by the inductance of a single coil, but at smaller separations the total sensor inductance is the combination of single coil inductance (Equation 2) and total mutual inductance (Equation 6).

(8) $$L=L_{\text{Coil}}(1+k)$$

This is unique relative to sensors with connected coils where the inductance at large separations is twice the inductance of a single coil.

### Total Capacitance

The total capacitance of the system is defined by a combination of single coil parasitic capacitances (CStray) and the capacitive interaction (parallel plate capacitance) between the two spiral coils (CPP). These two sources of capacitance are added to give the total capacitance as shown in Equation 9.

(9) $$C=C_{\text{Stray}}+C_{\text{pp}}$$

The parallel plate capacitance Cpp between the two coils can be modeled as:

(10) $$C_{\text{pp}}=(\varepsilon_{0}\varepsilon_{r}A_{\text{overlap}})/l$$

Where ε_r_ is the dielectric constant of air = 1, A_overlap_ is the area of conductor overlap between the coils as calculated by Equation 11 and l is the spacing between the coils.

(11) $$A_{\text{overlap}}=T_{W}\times {\text{length}}_{\text{trace}}$$

Where the length of the overlapping trace is approximated as:

(12) $${\text{length}}_{\text{trace}}\approx \pi (T_{W}+T_{S})/{\left[2T_{s}+2T_{w}\right]}^{2}\times {\left[D_{o}-2T_{s}\right)}^{2}-{\left(D_{i}-2T_{s}\right)}^{2}]$$

From Equations 10-12, the capacitive interaction between the two spiral coils can be calculated. The single coil parasitic capacitance arises from the turn to turn capacitance between adjacent turns within a single coil. We assume the turns to function like inter-digitated electrodes whose fringing fields are affected by the dielectric constants of the media on either side [19–21].

**Figure 4:**
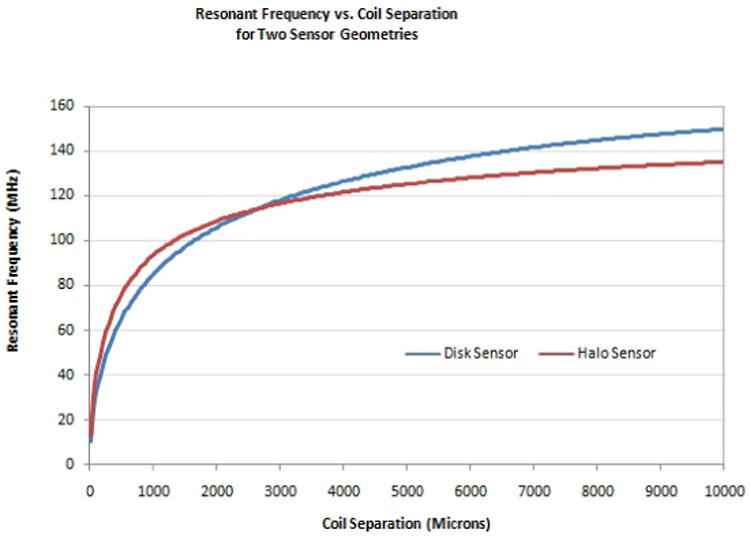
Resonant frequency is altered by coil separation. The change in frequency is governed by the geometric and dielectric properties of the sensor components.

The inductance and capacitive interaction between the two coils are derived in Equations 2-12. These values are calculated based on the geometric and dielectric properties of the sensor components. Stray capacitance also affects sensor performance, but must be determined experimentally. For the geometries of Table 1, the value of the stray capacitance for both the halo and disk coil configurations was experimentally measured as detailed below. Once measured, the total capacitance and total inductance values were substituted into Equation 1 which gives the theoretical resonant frequency for the system based on the geometric properties of the individual coils and the separation between them. For the two geometries defined in Table 1, the resonant frequency from Equations 1-12 of each coil is shown in Figure 4.

**Figure 5:**
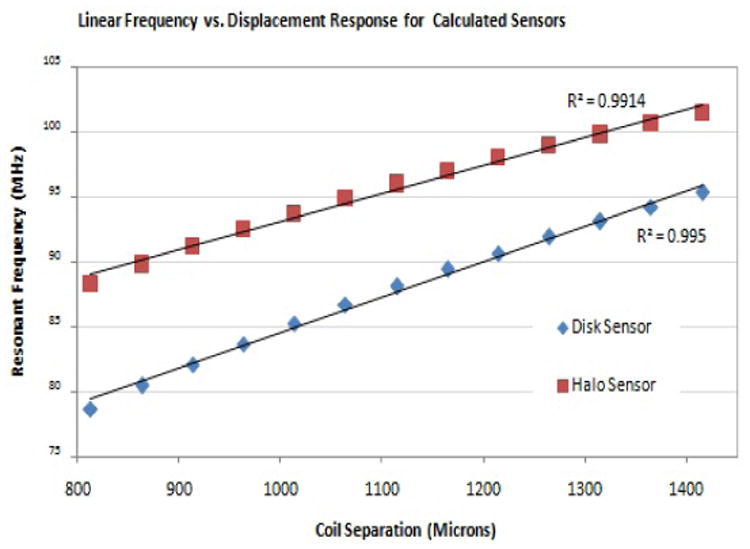
For each sensor geometry, there is a range of coil separation which results in a linear change in resonant frequency. Values predicted by the analytical model are plotted with a linear regression shown. The high correlation coefficients indicate a highly linear relationship within this range.

For the sensor configurations shown, the frequency response is approximately linear within defined ranges of coil separation. For both of the coil geometries shown in Figure 4, the frequency change in the 800 to 1,400 μm range can be modeled as linear with very small error, as shown in Figure 5. From Equations 1-12, sensors can easily be designed to operate within this limited range of coil separation. This linear change in frequency in response to changes in coil separation is unique and serves as the foundation for this novel class of passive wireless sensors.

For the sensor configurations shown, the frequency response is approximately linear within defined ranges of coil separation. For both of the coil geometries shown in Figure 4, the frequency change in the 800 to 1,400 μm range can be modeled as linear with very small error, as shown in Figure 5. From Equations 1-12, sensors can easily be designed to operate within this limited range of coil separation. This linear change in frequency in response to changes in coil separation is unique and serves as the foundation for this novel class of passive wireless sensors.

For the data shown in Figure 5, linear regression was used to define the first order relationship between coil separation and resonant frequency. The slope of the regression line describes the sensitivity of the sensor and the intercept defines the “resting” frequency at the lower end of the linear range. For the disk and halo sensors defined in Table 1 and shown in Figure 5, the linear relationships between frequency and coil separation are:

(13) $$f_{0}=0.0217(l)+71.4$$

(14) $$f_{0}=0.0275(l)+57.0$$

These simple relationships predict the performance of sensors within the linear range based on their specific geometric properties.

### Sensor Fabrication and Testing

Batch fabrication via photolithographic patterning and electroplating was used to produce matched pairs of Archimedean coils to test the performance of the sensor system. Both disk coils and halo coils were fabricated for evaluation and testing.

### Photolithographic Mask Design and Fabrication

**Figure 6:**
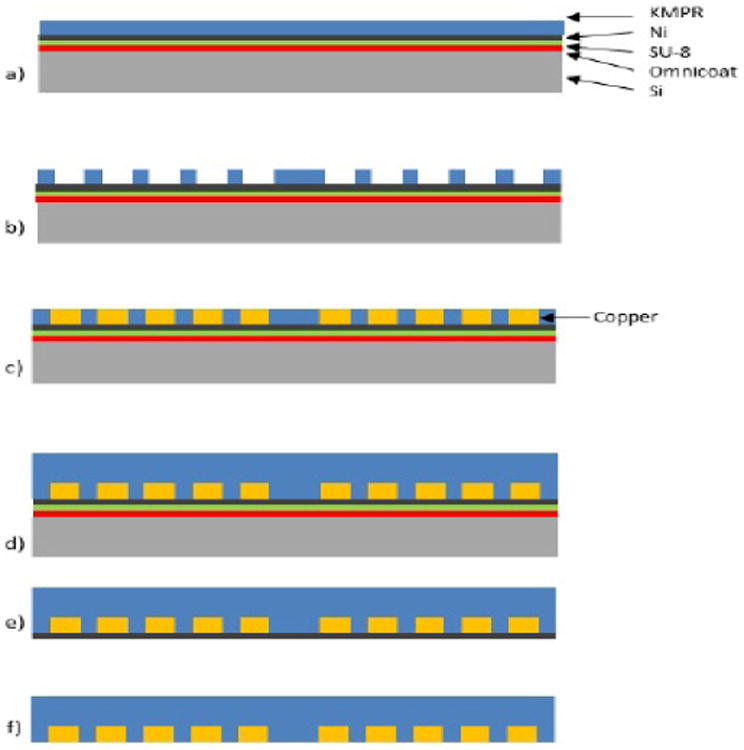
Copper coils were fabricated using photolithography and electroplating. a) The initial stack consists of the silicon wafer substrate, Omnicoat release layer, SU-8 release layer, nickel seed layer, and KMPR plating resist layer. b) The first round of photolithography defines the coils. c) The copper is electroplated using the nickel layer as a seed. d) The electroplated coils are coated with a capping layer of KMPR. e) The KMPR/coil layer is released from the silicon wafer via removal of the sacrificial layers. f) The nickel seed layer is removed to prevent electrical shorting of the coil windings.

Copper coils were fabricated using a combined approach of photolithography and metal electroplating. A series of photolithographic masks were designed to define the coils and also to define the secondary capping layer to protect the coils. Masks were designed in Layout Editor (Juspertor; Unterhaching, Germany) and fabricated on glass/chrome photomasks (Photosciences Inc.; Torrance, CA). A schematic of the steps used to fabricate the coils is shown in Figure 6. First, a 100 mm diameter silicon wafer was spin-coated with a layer of Omnicoat (MicroChem; Newton, MA) to approximately 20 nm thick, followed by baking at 200°C for 1 min. The wafer was then spin coated with SU-8 2010 (MicroChem; Newton, MA) to an approximate thickness of 10 μm. This provided a double-thick sacrificial release layer, for removal of the coils from the silicon wafer, post-electroplating. Following these steps, 150 nm of nickel was deposited onto the sacrificial layers using electron beam evaporation. The nickel surface was then cleaned with acetone and isopropanol and coated with KMPR plating resist (MicroChem; Newton, MA) to thicknesses ranging from 30 – 100 μm. The wafer was then baked at 100°C for at least 15 minutes.

Using a photomask, contact photolithography was then employed to form the coil structures. After exposure, the wafer was baked at 100°C to fully cross-link the exposed resist.

Loctite Hysol E-20HP (Henkel Corp.; Rocky Hill, CT) was then used to coat the outer radius of the silicon wafer, to protect the sacrificial release layers during development of the KMPR resist. Post-exposure development of unexposed KMPR was performed in SU-8 developer for 15 min. Using these steps, the coil structures were defined in the KMPR layer, exposing the nickel layer at the bottom of these structures. The nickel layer was then used as a seed for electroplating copper.

To fabricate the copper windings (coils) within the KMPR structures, solution-based copper electroplating was performed using a galvanostat (BioLogic SP-50; Grenoble France). The coils were plated such that the copper did not exceed the height of the KMPR “mold”. The current density and electroplating time was modulated such that only enough copper was plated to fill the molds to the desired height.

A copper electroplating solution consisting of 1.5 M sulfuric acid, 0.63 M copper sulfate, and 50 ppm hydrochloric acid was prepared from individual components for this process (Sigma-Aldrich; St. Louis, MO). Electrical contact was made to the exposed nickel layer (on the wafer), which likewise provided electrical contact to the entire nickel layer on the wafer. Following electroplating, a capping layer of KMPR was spin-coated onto the wafer, to protect the exposed copper from potential corrosion or mechanical damage. The resulting KMPR layer (100μm thickness) was then exposed using a second photolithographic mask to define the capping layer. The wafer was then post-exposure baked at 100°C for 15 min and developed in SU-8 developer. The wafer was then cleaved to expose the sacrificial layers, and then immersed in SU-8 developer until the nickel/KMPR/coil layer released from the silicon wafer.

The released coils were then immersed in nickel etchant to remove the nickel seed layer. Finally, the coils were rinsed in isopropanol and deionized water.

### Coil Testing

The resonant frequency of six individual coils was measured for both the halo and disk geometries to determine the stray capacitance of an individual coil (Equation 9). First, the inductance of a single coil was calculated from Equation 2 and this was substituted into Equation 1. The resonant frequency of the coil was then measured by recording the S11 parameter via a vector network analyzer (Agilent Technologies; Santa Clara, CA) and a manually fabricated antenna (4-turns, 1 cm diameter, fabricated from 24 gauge wire) to interface with the coil. From the measured resonant frequency and the calculated inductance from Equation 2, Equation 1 was used to solve for the capacitance. For a single coil, Cpp = 0 and so from Equation 9, C=C_Stray. In this way, the stray capacitance of a single coil back-calculated for use in Equation 9.

### Sensor Testing

**Figure 7:**
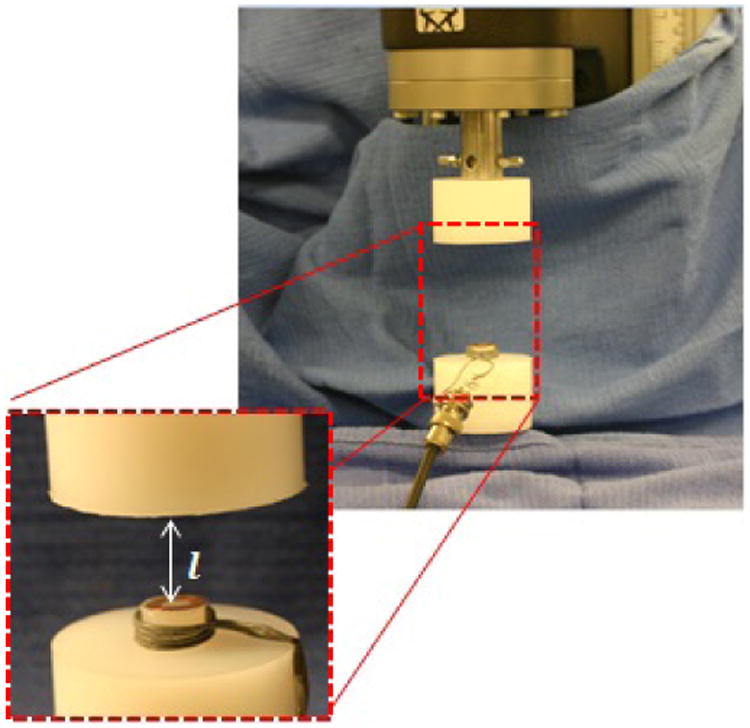
Each pair of sensors was tested on a high molecular weight polyethylene test fixture on a mechanical tester. The coils were moved apart incrementally to change the separation distance shown as l.

After fabricating batches of coils, we tested matched pairs of coils experimentally to validate the analytical model. The displacement-frequency relationship between the coils was measured experimentally by mounting anti-aligned coils (coils which are parallel and co-axial but with windings oriented in opposite directions) to high molecular weight polyethylene platens with double-sided tape as shown in Figure 7. The platens were placed in a mechanical testing machine (Instron; Norwood, MA) and the crosshead was used to incrementally change the coil separation.

**Figure 8:**
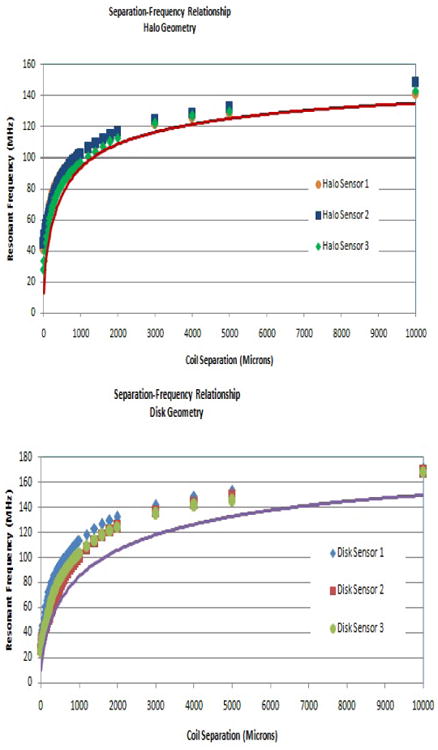
Each sensor was tested five times. Both halo and disk sensors demonstrated similar trends as coil separation increased. These changes were mirrored by the theoretical values, shown as a solid line. The error between the theoretical and the measured data is at a maximum at close coil displacements however the error steadily declines with increasing displacements. Each point on the graphs represents the mean of the five replicate measurements for each sensor. Error bars show the standard deviation.

At every interval, the resonant frequency was measured by monitoring the S11 parameter via a vector network analyzer and the same antenna described above. Six coil pairs were tested incrementally between 0-1 cm at increments ranging from 25 μm to 1,000 μm as shown in Figure 8. Each pair of coils was tested five times. The average of all five trials was compared to the theoretical curves.

### Sensor Calibration

Our sensors transduce a change in coil separation to a change in frequency. Frequency is monitored and from the relationships derived in Equations 1-12, the frequency can be used to back calculate the change in coil separation. Within each sensor's linear range, Equations 13 and 14 describe the linear relationship between coil separation and frequency. We conducted an experimental calibration of each sensor to refine this relationship and account for minor differences between individual sensors. Solving Equations 13 and 14 for coil separation yields:

(15) $$l(f_{0})=(f_{0}-71.4)/0.0217$$

(16) $$l(f_{0})=(f_{0}-57.0)/0.0275$$

A single frequency measurement was taken for each sensor at the lower limit of the defined linear range. The resonant frequency (f_cal) at that coil spacing distance (l_cal) was recorded. The values were used to make empirical corrections to the intercepts in Equations 13-16.

(17) $$\Delta l=(f_{\text{cal}}-71.4)/0.0217-l_{\text{cal}}$$

(18) $$\Delta l=(f_{\text{cal}}-57.0)/0.0275-l_{\text{cal}}$$

Where Δl is the empirical correction for each individual sensor. Using these corrections, the resonant frequency-coil spacing relationship in the linear range for any individual coil with the geometry found in Table 1 is described by:

(19) $$l(f_{0})=(f_{0}-71.4)/0.0217-\Delta l$$

(20) $$l(f_{0})=(f_{0}-57.0)/0.0275-\Delta l$$

Where Equations 19 and 20 are for the disk sensors and halo sensors, respectively. In this way, the performance of any wireless displacement sensor can be easily predicted using Equations 1-12 with a linear regression analysis and a single calibration measurement.

## Results

### Sensor Fabrication

Twenty-five coils each of the two geometries in Table 1 were fabricated using photolithography and electroplating. A current density of 10 mA/cm2 was applied for 131 minutes to plate copper for the 30 microns coils. From Equation 12, conductor lengths of 50.9 and 52.7 cm were calculated for halo and disk coils respectively. These conductor lengths were used to calculate the area of overlap in Equation 11. The area of overlap was 0.51 and 0.84 cm2 for halo and disk coils respectively. Both of these values were used for the calculation of parallel plate capacitances in Equation 10.

### Coil Testing

The resonant frequencies of the 6 coils per geometry were averaged for use in Equation 1. The single coil inductance was calculated using Equation 2 for each coil using the geometry from Table 1. The stray capacitance was calculated by solving for C in Equation 1 using the measured frequency and the calculated single coil inductance. For individual halo coils, the average measured resonant frequency was 145.2 MHz. The calculated inductance of these same coils was 3.62 μH. From these values, the stray capacitance was calculated to be 0.33 pF. For a single disk coil, the average measured resonant frequency was 170 MHz and the calculated inductance was 3.25 μH. The stray capacitance was calculated to be 0.27 pF. As expected, the halo geometry results in both a higher inductance and stray capacitance than the disk geometryas summarized in Table 2 [13,14].

### Sensor Testing

As expected, when the distance between the coils increases, both the halo and the disk sensors exhibit an asymptotic approach to the resonant frequency of a single coil. The resonant frequencies of the disk sensor were lower than the halo sensors at closer distances as predicted by theory (Figure 4). For all sensors tested, the theoretical frequency was lower than the measured frequency, however the displacement-frequency trends are well represented across all distance scales as shown in Figure 8.

### Sensor Calibration

**Figure 9:**
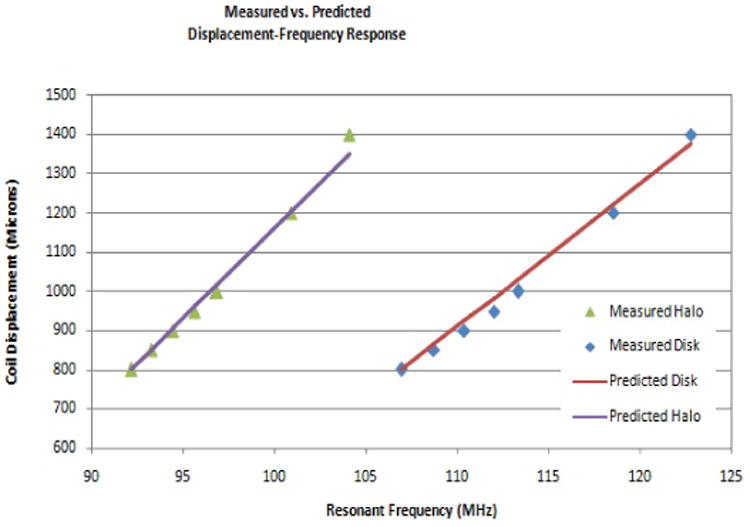
The linear model of both the halo and the disk sensors predicts experimental performance well. The linearization of the frequency-displacement relationship combined with the calibration procedure make the described protocol robust regardless of minor differences inresting frequencies.

When a linear best fit model was used to approximate the coil separation-frequency relationship in the defined linear range, correlation coefficients ranged from 0.9876 to 0.9958. Individual sensors were calibrated in this same range with l_calset at 800 μm. The halo and disk sensors exhibited sensitivities of 46 and 36 μm/MHz respectively. When comparing the linear model to experimental data, errors ranged from 1.1% to 2.7% for halo sensors and 2% to 5% for the disk sensors. Characteristic plots are shown in Figure 9 for a halo and a disk sensor.

## Discussion

We have developed a simple sensor system that transduces physical changes in displacement to a change in sensor resonant frequency. The sensor system is simple, inexpensive, and requires no onboard signal conditioning electronics. The sensor system is unique in that its change in frequency output is linear with respect to change in displacement through a designated range. This unique feature makes the sensors easy to predict, calibrate, and monitor.

Because the sensors are comprised only of two anti-aligned Archimedean coils, batch fabrication is also simple and inexpensive. The sensor components (coils) can be fabricated with photolithography and electroplating using well-established techniques. The fabrication technique is also amenable to making sensors in various shapes, sizes, and configurations for different applications.

Our data show that for two different configurations (disks and halos), we are able to predict the performance of the sensors accurately. Figure 8 and 9 demonstrate that our analytical model predicts sensor performance throughout its entire range. Importantly, we are able to use our model to determine the range within which the sensors respond linearly to a change in displacement. This greatly simplifies calibration and measurement.

The behavior of the batch fabricated sensors is predicted well by the analytical model. The simple calibration protocol further reduces errors between the model and experimental data. The errors (by percent) are greatest at small coil displacements where the analytical model underestimates the resonant frequency. This suggests that the model overestimates either L or C at short distances (Equation 1). The overestimation of L or C may be because the present model assumes that each conductive trace in one coil aligns with a conductive trace from the other coil. Because our coils are anti-aligned, this is not always the case. At larger coil displacements, the contribution of parallel plate capacitance is diminished and the errors are reduced as displacement increases. Through the empirical corrections described, the model is refined in a way that these errors are minimal as shown in Figure 9.

The sensors presented here are comprised of a pair of coils with air as the dielectric between. This configuration is sufficient to demonstrate the theory which governs the behavior of the system and to validate our analytical model. In practice, a solid dielectric will be interposed between the two coils. The solid dielectric bonds the coils to each other and changes the functionality of the system.

Uniquely, a sensor which is comprised of two coils with a solid dielectric between can act as a force sensor. When a load is applied normal to the surface of one of the coils, it compresses the dielectric and the spacing between the coils changes. The change in coil spacing is proportional to the mechanical properties of the dielectric. As the spacing between the coils changes, the resonant frequency of the sensor changes as described in Equations 1-12. The dielectric properties of the interposing solid material and the geometry of the coils govern the response. Using these relationships with the known mechanical properties of the solid dielectric, the measureable change in frequency is used to calculate the applied force. Thus, the sensors function as a simple wireless load cell with no electrical connections.

Likewise, if the solid dielectric has a Poisson's ratio of less than 0.5, the sensors can be designed to be sensitive to changes in hydrostatic pressure. As pressure changes, the dielectric deforms and coil spacing changes. In this way, the sensors function as a simple wireless pressure transducer with no electrical connections.

The sensor system described here is a simple, inexpensive, passive, wireless system that is small and robust. As such, it is amenable to applications in medicine where an implantable sensor is desired. Because of its small size, cost, and simplicity, it potentially obviates the need for modification of implants, introduces negligible increase in cost, and is simple enough that it is robust in the in vivo environment. Thus, the simple Archimedean spiral pairs with interposing dielectric have the potential to function as a linear passive wireless implantable sensor for many applications.

## Figures and Tables

**Table 1:** The geometry of each coil configuration dictates the properties of the resonant behavior of the senor. Using photolithography and electroplating, sensors can be fabricated in a range of geometries. Both disk and halo coils were fabricated and tested to validate the analytical model.

	Disk Coil	Halo Coil
Do-Outer Diameter (mm)	12	11
Di-Inner diameter (mm)	0	5.25
N-Number of Turns	28	19
T_H_-Trace Hight (μm)	30	30
T_W_-Trace Width (μm)	160	100
T_S_-Trace Spacing (μm)	50	50
I_H_-Insulator Hight (μm)	100	100

**Table 2:** The resonant frequency of individual coils was measured experimentally. The inductance of individual coils was calculated from the geometry of each coil. The stray capacitance of a single coil was calculated by solving for C in Equation 1 using the measured frequency and calculated inductance.

	Halo Coils	Disc Coils
f_0_ measured (MHz)	145	170
L calculated (μH)	3.62	3.252
C_S_traycalculated (pF)	0.333	0.27
